# Hyperintense Thyroid Incidentaloma on Time of Flight Magnetic Resonance Angiography

**DOI:** 10.3389/fendo.2018.00417

**Published:** 2018-07-23

**Authors:** Soo Chin Kim, Inseon Ryoo, Hye Young Sun

**Affiliations:** ^1^Department of Radiology, Seoul National University Hospital Healthcare System Gangnam Center, Seoul National University College of Medicine, Seoul, South Korea; ^2^Department of Radiology, Korea University Guro Hospital, Korea University College of Medicine, Seoul, South Korea

**Keywords:** time of flight magnetic resonance angiography, incidental findings, thyroid nodules, sonography, incidentaloma

## Abstract

**Background:** The purpose of this study was to evaluate the clinical significance of thyroid incidentaloma with hypersignal intensity on the time of flight magnetic resonance (TOF-MR) angiography and correlation with ultrasound (US).

**Methods:** We retrospectively reviewed 3,505 non-contrast TOF-MR angiography performed at our institution between September 2014 and May 2017. Two radiologists correlated the thyroid incidentalomas detected on TOF-MR angiography with US features that were obtained within a three-month interval between MR and US examinations in consensus.

**Results:** The prevalence of hyperintense thyroid nodules incidentally detected by TOF-MR angiography was 1.2% (43/3,505 patients). Among these, 35 people (77.8%) underwent US examinations, and a total of 45 hyperintense thyroid nodules were detected by US studies. Of these 45 nodules, more than 70% were categorized as benign on US exams. Fine needle aspiration was performed on nine nodules according to indications recommended by the Korean Society of Thyroid Radiology. All except one high-suspicion thyroid nodule were confirmed as benign (Bethesda 2) on cytologic examination. The high-suspicion nodule on US showed a nondiagnostic result (Bethesda 1). However, this nodule collapsed after aspiration of thick colloid.

**Conclusions:** Our study demonstrated that the most hyperintense thyroid nodules detected on TOF-MR angiography were benign. Therefore, if a hyperintense incidentaloma is found on TOF-MR angiography, the thyroid nodule is more likely to be benign. We believe that these findings could offer additional information for further clinical management.

## Introduction

Thyroid nodules are very common. An autopsy study of 821 consecutive patients with clinically normal thyroid glands found the prevalence of thyroid nodules to be greater than 50% (1). A thyroid incidentaloma is an unsuspected thyroid nodule found in the course of examinations performed for other reasons in a person without clinical symptoms or suspicion of thyroid nodule. With advances in and commercialization of medical imaging, thyroid incidentalomas are increasingly reported. Prospective studies in the general population show a high prevalence of small thyroid nodules measuring < 10 mm in 70–83% of patients receiving ultrasonography (US) (2, 3). The prevalence is lower on computed tomography (CT) or magnetic resonance imaging (MRI) of the neck, at around 16%, and with fluorodeoxyglucose (FDG) positron emission tomography/computed tomography (PET/CT), at around 5% (4).

Time of flight (TOF)-MR angiography is a widely used, noninvasive method for evaluating brain and neck vessels without contrast agent. We occasionally observe focal high signal intensity lesions within the thyroid glands as thyroid incidentalomas in daily practice. We found three reports about the prevalence and radiologic correlation of thyroid incidentalomas found on MR angiography (5–7); all used MR angiography with contrast administration.

Therefore, the purpose of our study was to evaluate the prevalence of thyroid incidentalomas detected by noncontrast TOF-MR angiography as a focal hyperintensity and to evaluate their clinical significance by correlation with US findings.

## Materials and methods

From September 2014 to May 2017, 3,505 asymptomatic people (2,073 male, 1,432 female; mean ± SD age, 57.3 ± 10.4 years; range, 20–82 years) underwent noncontrast neck TOF-MR angiography examinations for health screening at our health care center.

### TOF-MR angiography

All MR examinations used 1.5T or 3T scanners (Espree, Achieva; Philips, Best, Netherlands and Skyra; Siemens, Erlangen, Germany) using standard clinical protocols of noncontrast TOF MR angiography techniques. MR parameters were: Espree, 31/7 ms (TR/TE), 384 × 200 matrix, 199 × 160 mm FOV, 25° FA, 1 mm slice thickness; Achieva, 24/4.2 ms (TR/TE), 304 × 174 matrix, 150 × 150 mm FOV, 22° FA, 2.6 mm slice thickness; and Skyra, 20/3.3 ms (TR/TE), 384 × 139 matrix, 240 × 145 mm FOV, 15° FA, 1.0 mm slice thickness. To reduce saturation effects, tilted optimized nonsaturating excitation/ramped pulse was applied to all MR angiography studies. Neck TOF-MR angiography was acquired in transverse planes and extended to the aortic arch and adjacent parts including thyroid glands. Source images were generated using steady-state coherent gradient echo sequences, and post processing was performed using a maximum intensity projection. Both were displayed on picture archiving and communication systems.

### Image analysis of MR angiography

Increased signal intensity (SI) was defined as a high-intensity area in contrast to the adjacent thyroid parenchyma on source images of neck TOF-MR angiography. All positive images were identified by a head and neck neuroradiologist (SCK with seven years of experience) and subsequently confirmed in consensus with a second head and neck neuroradiologist (IR with seven years of experience). The location (right, left, and isthmus) and extent of hyperintense portions within the nodule were evaluated as partial (≤ 50%, if the hyperintense lesion occupied less or equal to 50% of the delineable thyroid nodule), predominant (50% < hyperintensity ≤ 70%), and total (hyperintensity >70%). Size as the largest diameter of the hyperintense nodule and normalized SI (nSI) were also measured. The nSI was calculated as SI of the hyperintense portion within the thyroid nodule/SI of contralateral normal thyroid parenchyma (ROI = 30 mm^2^).

### US examination and image analysis

Of participants with hyperintense thyroid nodules viewed on neck TOF-MR angiography, we selected those who underwent thyroid US examination for thyroid screening within a 3-month interval between MR and US studies. All US examinations used iU 22 systems (Philips Medical Systems, Bothell, WA, USA; 5–12 MHz linear probe) or Logiq E9 (GE Medical Systems, Milwaukee, WI, USA; 6–15 MHz linear probe). Two head and neck radiologists retrospectively and by consensus reviewed US images of thyroid nodules that corresponded to the hyperintense nodule on neck TOF-MR angiography. First, the largest diameters of the thyroid nodules were measured. Then, we evaluated the internal contents of thyroid nodules as solid (no obvious cystic content), predominantly solid (≤ 50% of the cystic portion), predominantly cystic (> 50% of the cystic portion), or cystic (no obvious solid content) and categorized nodules according to the Korean Thyroid Imaging, Reporting, and Data system (K-TIRADS); ACR Thyroid Imaging, Reporting, and Data System (ACR-TIRADS); and European Thyroid Imaging, Reporting, and Data System (EU-TIRADS) classifications (8–10). For patients who underwent fine-needle aspiration biopsy (FNAB), cytologic diagnoses were reported according to the Bethesda System (six categories). In patients with follow-up US examination during the study period, follow-up imaging findings were also evaluated.

### Laboratory examinations

Laboratory examinations including thyroid function test and thyroid antibody test were recorded.

### Statistical analysis

The sizes measured on TOF-MR angiography and US were compared using paired *t*-tests. The extent of the hyperintense portion of nodules and nSI of thyroid nodules on TOF-MR angiography according to internal contents and US classification categories (K-TIRADS, ACR-TIRADS, EU-TIRADS) on US were assessed using ANOVA and Kruskal-Wallis tests. The Mann-Whitney test for comparisons between pairs of groups was performed when appropriate. Bonferroni's correction was applied to the post hoc analysis of between group comparisons to adjust for the number of comparisons performed. All statistical analyses were performed using IBM SPSS ver. 19.0 (IBM Co., Armonk, NY, USA). *P* < 0.05 was considered statistically significant.

## Results

**Figure 1:**
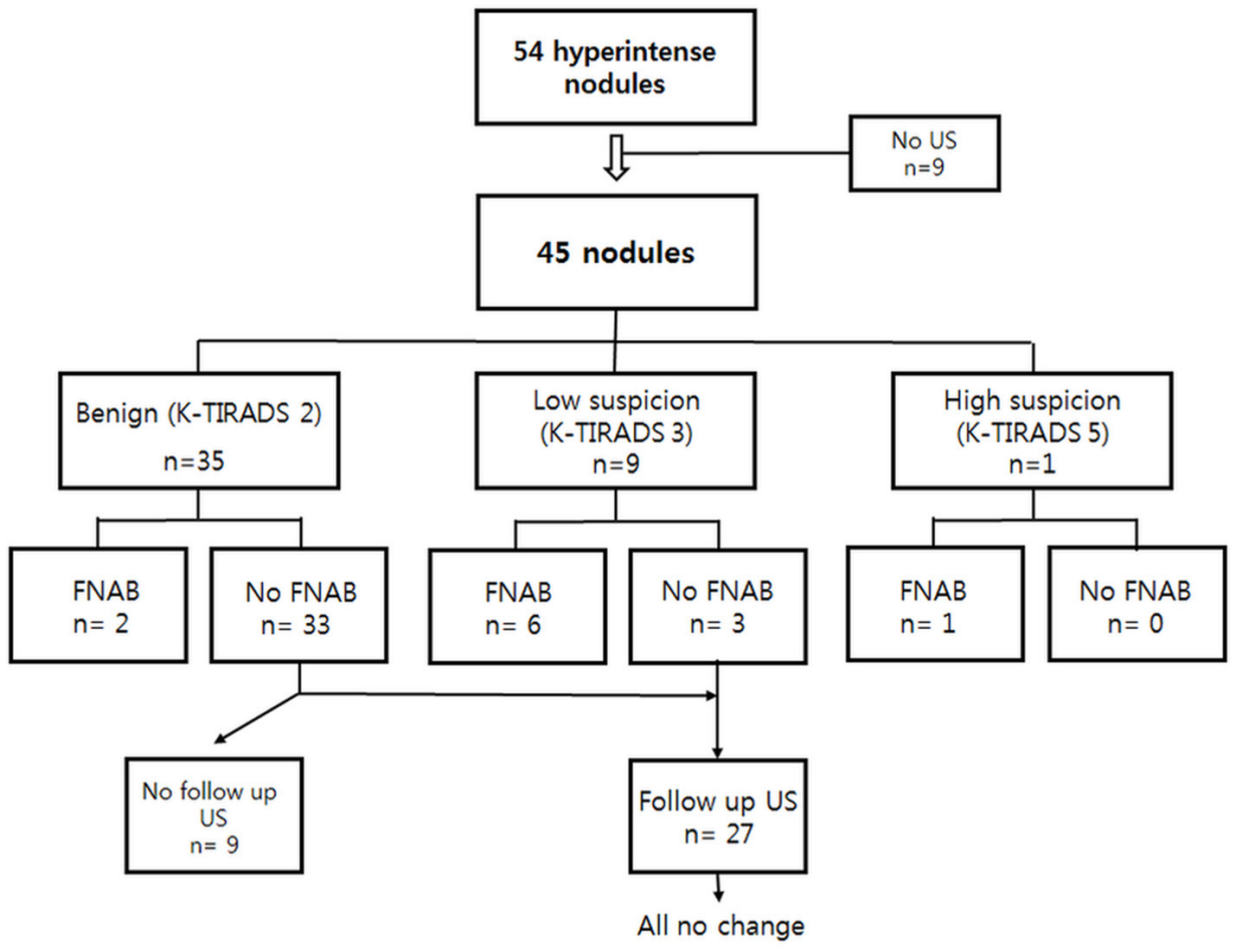
US assessment and US-guided FNAB results of 45 thyroid incidentalomas on TOF-MR angiography, followed by US. US, ultrasound; FNAB, fine needle aspiration biopsy; TOF-MR, time-of-flight magnetic resonance.

A total of 54 thyroid incidentalomas were detected on unenhanced TOF-MR angiography as focal hyperintensities in 43 people (17 men, 26 women; mean ± SD age, 59.4 ± 9.3 years; range, 31–76 years) among the 3,505 individuals who underwent TOF-MR angiography. The prevalence of hyperintense thyroid incidentalomas detected by noncontrast TOF-MR angiography was 1.2% (43/3,505 people). One hyperintense thyroid nodule was detected in 36 individuals, two nodules in four, three nodules in two, and four nodules in one person. Of the nodules, 25 were in the right lobe, two in the isthmus, and 27 in the left lobe. Among 43 people, nine did not undergo US. The remaining 45 incidentalomas in 34 people were detected by US (Figure 1). The mean time interval between MR angiography and US was 8 ± 39 days.

**Figure 2:**
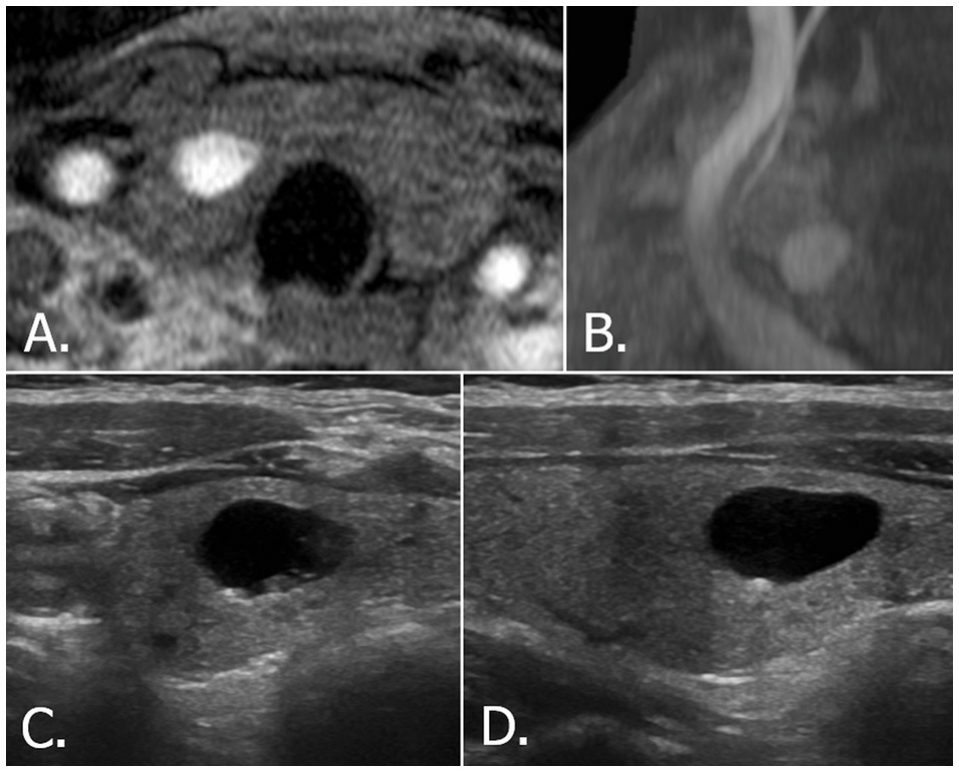
68-year-old female with right thyroid nodule. Source **(A)** and MIP **(B)** images of TOF-MR angiography show a totally hyperintense nodule in the right thyroid gland. Transverse **(C)** and longitudinal **(D)** US images of right thyroid reveals a smooth, oval, pure cystic nodule with comet tail artifact, which is classified as benign nodule (K-TIRADS category 2, ACR-TIRADS category 1 and EU-TIRADS category 2). MIP, maximum intensity projection; TOF-MR, time-of-flight magnetic resonance; US, ultrasound; K-TIRADS, Korean Thyroid Imaging Reporting and Data System; ACR-TIRADS, ACR Thyroid Imaging Reporting and Data System; EU-TIRADS, European Thyroid Imaging Reporting and Data System.

**Figure 3:**
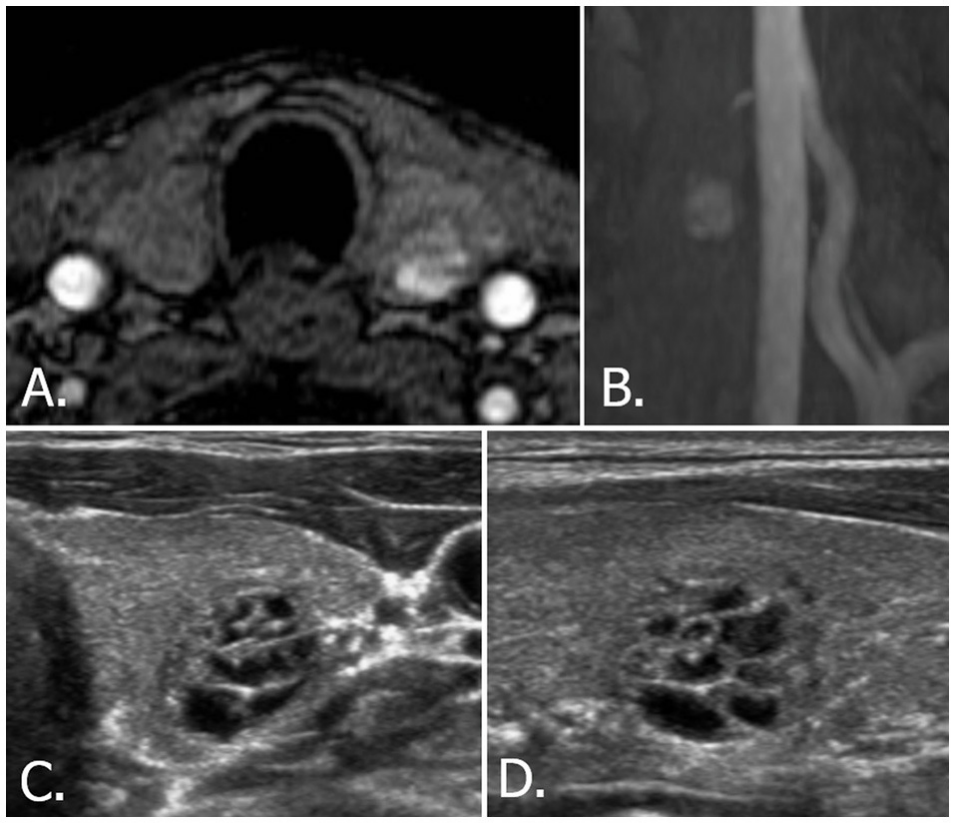
53-year-old male with left thyroid nodule. Source **(A)** and MIP **(B)** images of TOF-MR angiography show a totally hyperintense nodule in the left thyroid gland. Transverse **(C)** and longitudinal **(D)** US images of left thyroid reveals a spongiform nodule, which is classified as benign nodule (K-TIRADS category 2, ACR-TIRADS category 1, and EU-TIRADS category 2). MIP, maximum intensity projection; TOF-MR, time-of-flight magnetic resonance; US, ultrasound; K-TIRADS, Korean Thyroid Imaging Reporting and Data System; ACR-TIRADS, ACR Thyroid Imaging Reporting and Data System; EU-TIRADS, European Thyroid Imaging Reporting and Data System.

**Figure 4:**
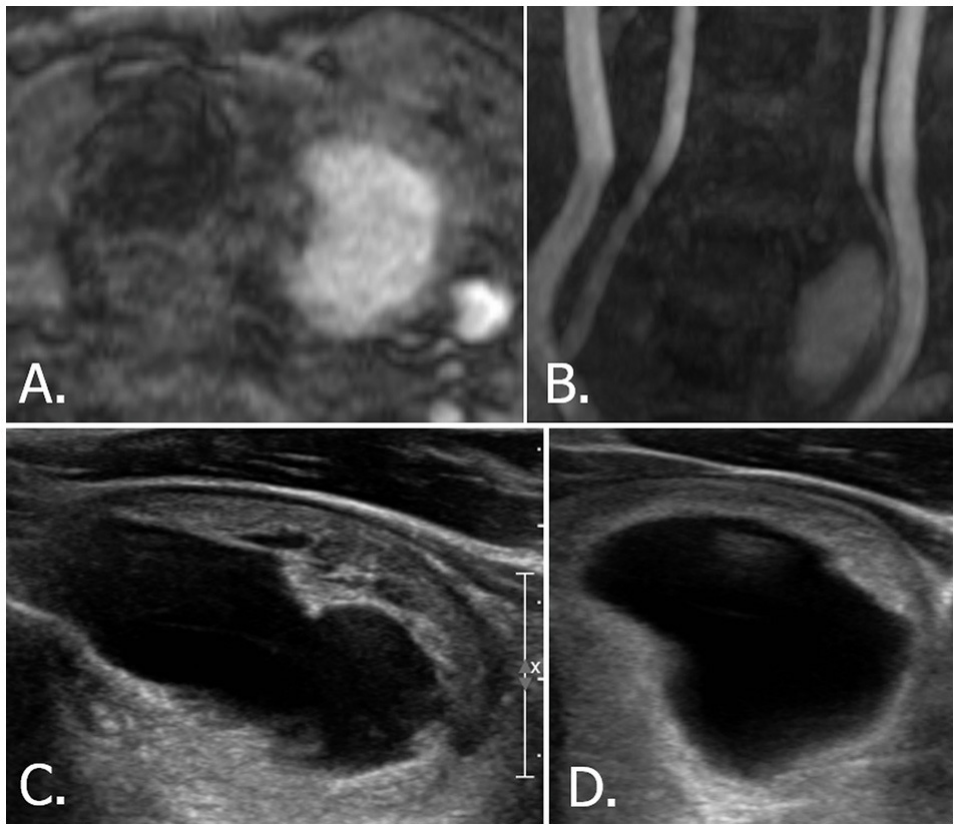
58-year-old male with left thyroid nodule. Source **(A)** and MIP **(B)** images of TOF-MR angiography show a totally hyperintense nodule in the left thyroid gland. Longitudinal **(C)** and transverse **(D)** US images of left thyroid reveals a smooth oval, predominantly cystic nodule with eccentric solid portion, which is classified as K-TIRADS category 3, ACR-TIRADS category 2, and EU-TIRADS category 3. MIP, maximum intensity projection; TOF-MR, time-of-flight magnetic resonance; US, ultrasound; K-TIRADS, Korean Thyroid Imaging Reporting and Data System; ACR-TIRADS, ACR Thyroid Imaging Reporting and Data System; EU-TIRADS, European Thyroid Imaging Reporting and Data System.

**Figure 5:**
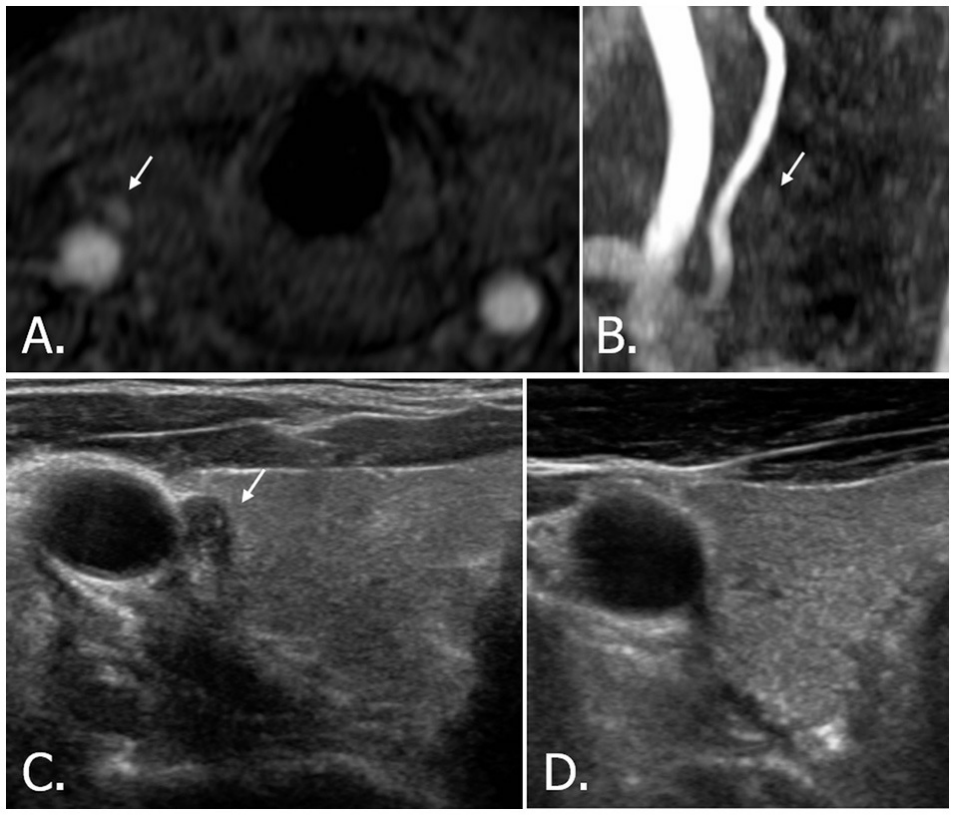
42-year-old male with right thyroid nodule. Source image **(A)** of TOF-MR angiography shows a small hyperintense nodule (white arrow) in the right thyroid gland. But the nodule (white arrow) is hardly detectable on MIP image **(B)**. Transverse **(C)** US image of right thyroid demonstrates a hypoechoic, solid nodule (white arrow) with echogenic spots, which is classified as highly suspicious nodule (K-TIRADS, ACR-TIRADS, and EU-TIRADS category 5). A cytologic result showed insufficient cellularity, but the nodule disappeared after aspiration **(D)**. TOF-MR, time-of-flight magnetic resonance; MIP, maximum intensity projection; US, ultrasound; K-TIRADS, Korean Thyroid Imaging Reporting and Data System; ACR-TIRADS, ACR Thyroid Imaging Reporting and Data System; EU-TIRADS, European Thyroid Imaging Reporting and Data System.

The mean largest diameter for hyperintense nodules from TOF-MR angiography was 0.88 ± 0.43 cm (range, 0.3–2.2 cm), which was significantly smaller than matched thyroid nodules on US images (1.41 ± 0.83 cm; range, 0.5–3.5 cm) (*p* = 0.00). Mean nSI was 1.93 ± 0.54 (range, 1.2–4.0). In 26 of 45 incidentalomas (57.8%), high signal intensity portions occupied more than 70% of the delineable thyroid nodule on TOF-MR angiography images (Figure 2). More than half of incidentalomas (29/45, 64.4%) were classified as pure cysts or predominantly cystic nodules, and more than 70% of nodules were categorized as benign (K-TIRADS 2, *n* = 35; ACR-TIRADS 1, *n* = 37; and EU-TIRADS 2, *n* = 33) on US exams (Figure 3).While there was no statistically significant difference with regard to extent of the hyperintense portion of nodules on TOF-MR angiography and US categories (*p* ≠0.05, Figures 3–5), the internal contents between the partial group and total groups showed a statistically significant difference based on the results of Bonferroni correction (*p* < 0.001). US results of thyroid incidentalomas detected on TOF-MR angiography are presented in Table 1. The nSI was not significantly different according to content of thyroid nodules or US category (*p* = 0.38 and 0.15, Figures 3–5) (Table 2).

**Table 1:** The extent of hyper signal intensity portion of thyroid nodules on TOF-MR angiography according to US features.

**US features**	**Extent of hyper signal intensity on TOF-MR angiography**			
	Parial (< 50%)^*^n = 13	**Predominant (50 ≤ < 70%), *n* = 6**	Total (≥70%)^*^n = 26	***p*-value^#^**
**Internal contents**				<**0.001**
Solid	0 (0%)	0 (0%)	1 (3.8%)	
P-solid	10 (76.9%)	3 (50%)	2 (7.7%)	
P-cystic	3 (22.1%)	3 (50%)	12 (46.2%)	
Cystic	0 (0%)	0 (0%)	11 (42.3%)	
**K-TIRADS category**				**0.051**
C2	7 (53.8%)	6 (100%)	22 (84.6%)	
C3	6 (46.2%)	0 (0%)	3 (11.6%)	
C5	0 (0%)	0 (0%)	1 (3.8%)	
**ACR-TIRADS category**				**0.145**
C1	9 (69.2%)	5 (83.3%)	23 (88.5%)	
C2	2 (15.4%)	1 (16.7%)	2 (7.7%)	
C3	2 (15.4%)	0 (0%)	0 (0%)	
C5	0 (0%)	0 (0%)	1 (3.8%)	
**EU-TIRADS category**				**0.060**
C2	7 (53.8%)	5 (83.3%)	22 (84.6%)	
C3	6 (46.2%)	1 (16.7%)	3 (11.6%)	
C5	0 (0%)	0 (0%)	1 (3.8%)	

* *The difference between partial group and total group is statistically significant (P < 0.05)*.

# *p-value for ANOVA and Kruskal-Wallis tests*.

*TOF-MR, time-of-flight magnetic resonance; US, ultrasound; P-Solid, predominantly solid; P-cystic, predominantly cystic; K-TIRADS, Korean Thyroid Imaging Reporting and Data System; ACR-TIRADS, ACR Thyroid Imaging Reporting and Data System; EU-TIRADS, European Thyroid Imaging Reporting and Data System*.

**Table 2:** The normalized SI on TOF-MR angiography according to US features.

**US features**	**Normalized SI**	***p*-value^#^**
**Internal contents**		**0.38**
Solid	1.59	
P-solid	1.77	
P-cystic	1.96	
Cystic	2.11	
**K-TIRADS category**		**0.15**
C2	2.01	
C3	1.66	
C5	1.59	
**ACR-TIRADS category**		**0.40**
C1	1.97	
C2	1.81	
C3	1.56	
C5	1.59	
**EU-TIRADS category**		**0.31**
C2	2.00	
C3	1.72	
C5	1.59	

# *p-value for ANOVA and Kruskal-Wallis tests*.

*SI, signal intensity; TOF-MR, time-of-flight magnetic resonance; US, ultrasound; P-Solid, predominantly solid; P-cystic, predominantly cystic; K-TIRADS, Korean Thyroid Imaging Reporting and Data System; ACR-TIRADS, ACR Thyroid Imaging Reporting and Data System; EU-TIRADS, European Thyroid Imaging Reporting and Data System*.

**Figure 6:**
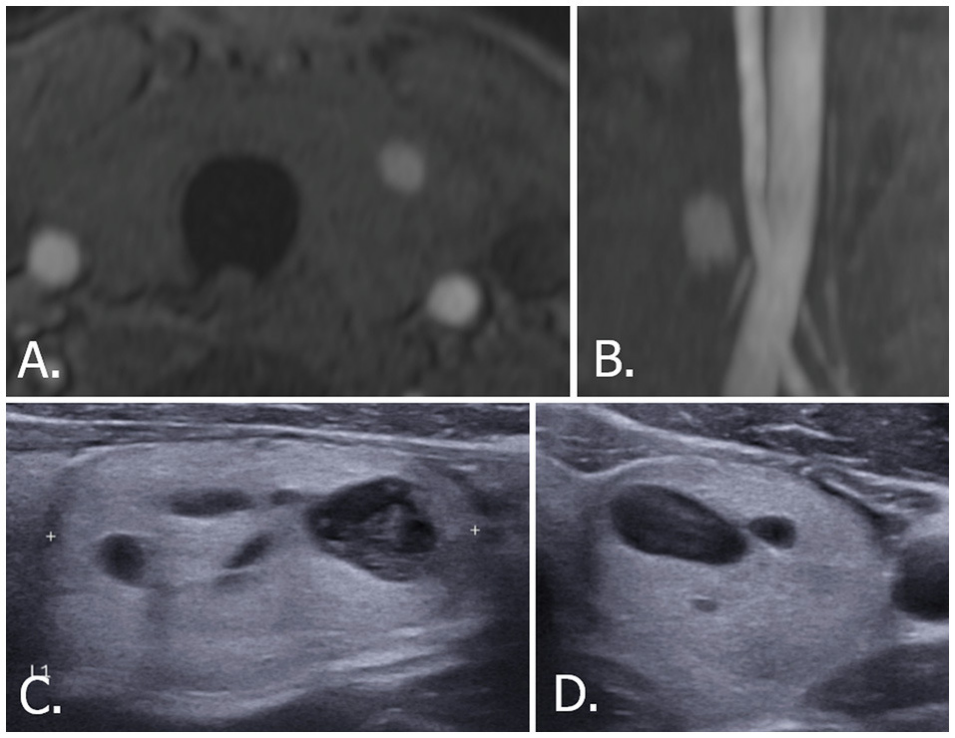
70-year-old male with left thyroid nodule. Source **(A)** and MIP **(B)** images of TOF-MR angiography show a nodule with hyperintense portion in the left thyroid gland. Transverse **(C)** and longitudinal **(D)** US images of left thyroid demonstrate a smooth, oval, isoechoic, predominantly solid nodule, which is classified as K-TIRADS category 3, ACR-TIRADS category 2, and EU-TIRADS category 3. FNA cytologic examination revealed a benign result. The hyperintense portion on TOF-MR angiography corresponds to the cystic portion of the nodule on US images. MIP, maximum intensity projection; TOF-MR, time-of-flight magnetic resonance; US, ultrasound; K-TIRADS, Korean Thyroid Imaging Reporting and Data System; ACR-TIRADS, ACR Thyroid Imaging Reporting and Data System; EU-TIRADS, European Thyroid Imaging Reporting and Data System; FNA, fine needle aspiration.

US-guided FNAB was performed on nine nodules according to indications recommended by the Korean Society of Thyroid Radiology (8): six thyroid nodules were categorized as low suspicion (K-TIRADS 3, Figure 6) and measured more than 1.5 cm, two thyroid nodules were categorized as benign (K-TIRADS 2) and measured more than 2 cm, and one thyroid nodule was categorized as high suspicion (K-TIRADS 5, Figure 5). These nine aspirated nodules included three nodules that were recommended for aspiration by the ACR (one category 3 thyroid nodule that measured more than 2.5 cm) or ETA (three category 3 nodules that measured more than 2 cm) recommendations.

All except one high suspicion thyroid nodule were confirmed as benign (Bethesda 2) on cytologic exams. One nodule with hyperechoic dots suggestive of microcalcifications on US (category 5 on K-TIRADS, ACR-TIRADS, and EU-TIRADS) showed a nondiagnostic result (Bethesda 1). However, this nodule collapsed after aspiration of thick colloid. Of the remaining 36 thyroid nodules, 27 that did not meet FNAB indications, including three K-TIRADS 3 nodules, were followed up. Of these, three thyroid nodules showed decreases in size and the others showed no significant interval changes on follow-up US taken at least 1 year later. Thyroid function and thyroid antibody test results for the 43 patients were normal.

## Discussion

TOF-MR angiography produces gradient-echo T1-weighted images with very short TR. When rapidly applied radiofrequency pulses are repeated at a region, stationary spins in that region cannot relax between pulses, and any background signal arising from these stationary spins degrades TOF data, eventually resulting in effective suppression of background signals except moving spins newly entering the section, such as blood. In spite of background suppression, lesions or materials with extremely short T1 relaxation time (i.e., thrombus, subacute hemorrhage, and melanin) can unintentionally be showed on TOF images (11). Therefore, thyroid nodules with such internal contents can be visualized on TOF-MR angiography.

We found that the prevalence of hyperintense nodules within the thyroid parenchyma by TOF-MR angiography was 1.2%. The prevalence of thyroid incidentalomas by MR angiography in our study was lower than that found in three previous studies, which reported 7.8, 5, and 4.6%, respectively (5–7). This difference could be due to the use of contrast-enhanced MR angiography in previous studies, which demonstrate both enhancing and non-enhancing thyroid nodules. Our study revealed only high-SI thyroid nodules without contrast.

The mean largest diameter of hyperintense nodules on TOF-MR angiography was smaller than the matched thyroid nodules on US images in our study. A prior study reported that non-sonographic cross-sectional imaging including CT, MRI, or PET-CT was more likely to underestimate nodule size compared with US, with a mean error 4.7 mm (12). In the present study, a mean error in cases of underestimation was larger than that of the prior study; we assumed that only the part of the thyroid nodule responsible for high signal intensity was noticeable, while other portions became undetectable because of background suppression. In the current study, 44 of 45 nodules were thought to have a cystic portion on US images. Only one was regarded as a solid nodule and was finally identified as a pure cyst upon aspiration. In addition, more than 70% of incidentalomas were regarded as benign on US classifications, because they exhibited cystic components with comet tail artifacts or a spongiform appearance on US images. The cystic portion of the thyroid nodule on US images tended to correlate with the extent of the hypersignal intense area on TOF images. The nSI seemed to increase as the cystic portion increased and was inversely correlated with K-TIRADS category, although without statistical significance. Six thyroid incidentalomas that lacked findings for colloid material were all confirmed as benign by FNAB. Therefore, we believe that colloid material or other proteins of degenerating cystic nodules can result in high T1 signal intensity on TOF images.

All thyroid incidentalomas in our study were regarded as sonographically benign or confirmed as benign by FNAB. Based on this finding and because thyroid cancers usually appear solid (13), we propose that incidentally detected thyroid cancers as hyperintensities on TOF-MR angiography images are rare. A previous study about MR features of thyroid tumors reported that most malignant thyroid tumors demonstrate isointense signals compared to normal thyroid tissue on T1-weighted images (14–17). Furthermore there were two patients with papillary thyroid carcinoma who underwent TOF-MR angiography and thyroid US within a 3-month interval between the two examinations before thyroidectomy in our institution in the same time period as this study. Those two papillary thyroid carcinomas also showed isointense signal on TOF-MR angiography (Supplementary Figures 1, 2). Therefore, readers couldn't detect the nodules on TOF-MR angiography and those were not included in this study. However, thyroid cancers can have cystic changes resulting from tumor necrosis or hemorrhage (18, 19), and these might cause T1 hyperintensity. However, we assume that hyperintensities of thyroid cancer would be difficult to visualize on TOF images. A previous study (20) found that most of the cystic portions of metastatic cervical lymphadenopathies from thyroid cancer, which have a similar mechanism to primary thyroid cancer, appeared to be hypointense lesions relative to fat.

In addition to the intrinsic limitations of selection bias from any retrospective study, our study had some limitations. First, the number of cases was rather small to generalize the results. Although we reviewed more than 3,500 examples of MR data, we found only 45 thyroid nodules because of the very low prevalence of thyroid incidentalomas on TOF-MR angiography. Further study with more cases is warranted to generalize these results. Second, cytologic results were obtained in just nine cases. Biopsies of nodules that do not meet the indication for FNAB are unethical. Furthermore, more than 70% of nodules without cytologic results in our study were followed, and none demonstrated increase in size on follow-up US performed at least 1 year later.

MRI examinations do not specify characteristics of thyroid incidentalomas that may be suspicious for cancer. A three-tiered system has been proposed for specific guidance to evaluate thyroid incidentalomas detected with CT, MRI, and PET. The system uses patient age, nodule size, and certain imaging features deemed to indicate high risk for malignancy (4, 21). However, our study showed that most hyperintense thyroid nodules detected on TOF-MR angiography were benign. Therefore, if a hyperintense incidentaloma is found on TOF-MR angiography, it is likely to be benign. This can potentially offer additional information for clinical management.

Recognition of these findings can form the basis of any clinical decision regarding further evaluations and management. If a hyperintense incidentaloma is found on TOF-MR angiography, further evaluation, especially invasive procedures of the nodules, could be suspended in a close conversation between patient and physician unless the patient shows other symptoms or clinical factors associated with thyroid nodules.

## Ethics statement

Informed consent was waived for all study participants due to the retrospective analysis, and the study protocol was approved by the Seoul national university hospital review boards.

## Author contributions

SCK and IR: Concept and design; SCK and IR: Analysis and interpretation of data, Manuscript writing, Review of final manuscript; All authors: Acquisition of data, Literature review, Refinement of manuscript.

### Conflict of interest statement

The authors declare that the research was conducted in the absence of any commercial or financial relationships that could be construed as a potential conflict of interest. The reviewer IR and handling Editor declared their shared affiliation.

## Supplementary material

Supplementary Figure 1 Thirty-Nine-year-old female with right papillary thyroid carcinoma. Source **(A)** and MIP **(B)** images of TOF-MR angiography show no hypersignal intense nodule in the right thyroid gland. Transverse **(C)** and longitudinal **(D)** US images of right thyroid gland demonstrates a hypoechoic solid nodule with microcalcifications which is classified as highly suspicious nodule (K-TIRADS, ACR-TIRADS, and EU TIRADS category 5). MIP, maximum intensity projection; TOF-MR, time-of-flight magnetic resonance; US, ultrasound; K-TIRADS, Korean Thyroid Imaging Reporting and Data System; ACR-TIRADS, ACR Thyroid Imaging Reporting and Data System; EU-TIRADS, European Thyroid Imaging Reporting and Data System; FNA, fine needle aspiration.

Supplementary Figure 2 Fifty-Two-year-old female with left papillary thyroid carcinoma. Source **(A)** and MIP **(B)** images of TOF-MR angiography show no hypersignal intense nodule in the left thyroid gland. Transverse **(C)** and longitudinal **(D)** US images of left thyroid gland demonstrates a markedly hypoechoic solid nodule with irregular/speculated margins which is classified as highly suspicious nodule (K-TIRADS, ACR-TIRADS, and EU TIRADS category 5). MIP, maximum intensity projection; TOF-MR, time-of-flight magnetic resonance; US, ultrasound; K-TIRADS, Korean Thyroid Imaging Reporting and Data System; ACR-TIRADS, ACR Thyroid Imaging Reporting and Data System; EU-TIRADS, European Thyroid Imaging Reporting and Data System; FNA, fine needle aspiration.
