# Supplementary figures and images for: Hyperintense Thyroid Incidentaloma on Time of Flight Magnetic Resonance Angiography
Source: Front Endocrinol (Lausanne). 2018 Jul 23;9:417. doi: 10.3389/fendo.2018.00417 (PMC6064725; doi:10.3389/fendo.2018.00417)

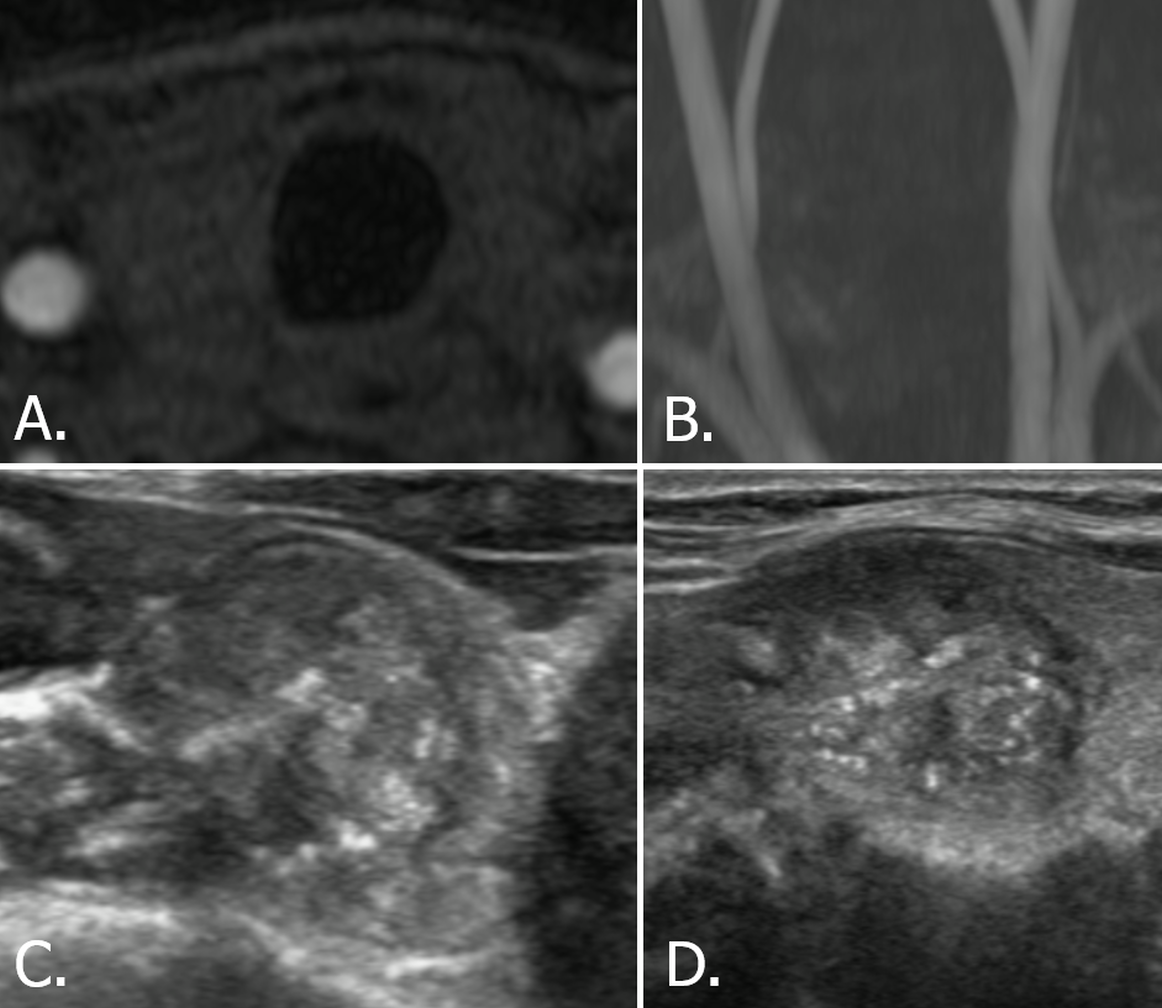

Supplement: Supplementary Figure 1 — Thirty-Nine-year-old female with right papillary thyroid carcinoma. Source (A) and MIP (B) images of TOF-MR angiography show no hypersignal intense nodule in the right thyroid gland. Transverse (C) and longitudinal (D) US images of right thyroid gland demonstrates a hypoechoic solid nodule with microcalcifications which is classified as highly suspicious nodule (K-TIRADS, ACR-TIRADS, and EU TIRADS category 5). MIP, maximum intensity projection; TOF-MR, time-of-flight magnetic resonance; US, ultrasound; K-TIRADS, Korean Thyroid Imaging Reporting and Data System; ACR-TIRADS, ACR Thyroid Imaging Reporting and Data System; EU-TIRADS, European Thyroid Imaging Reporting and Data System; FNA, fine needle aspiration. [file Image_1.TIF]

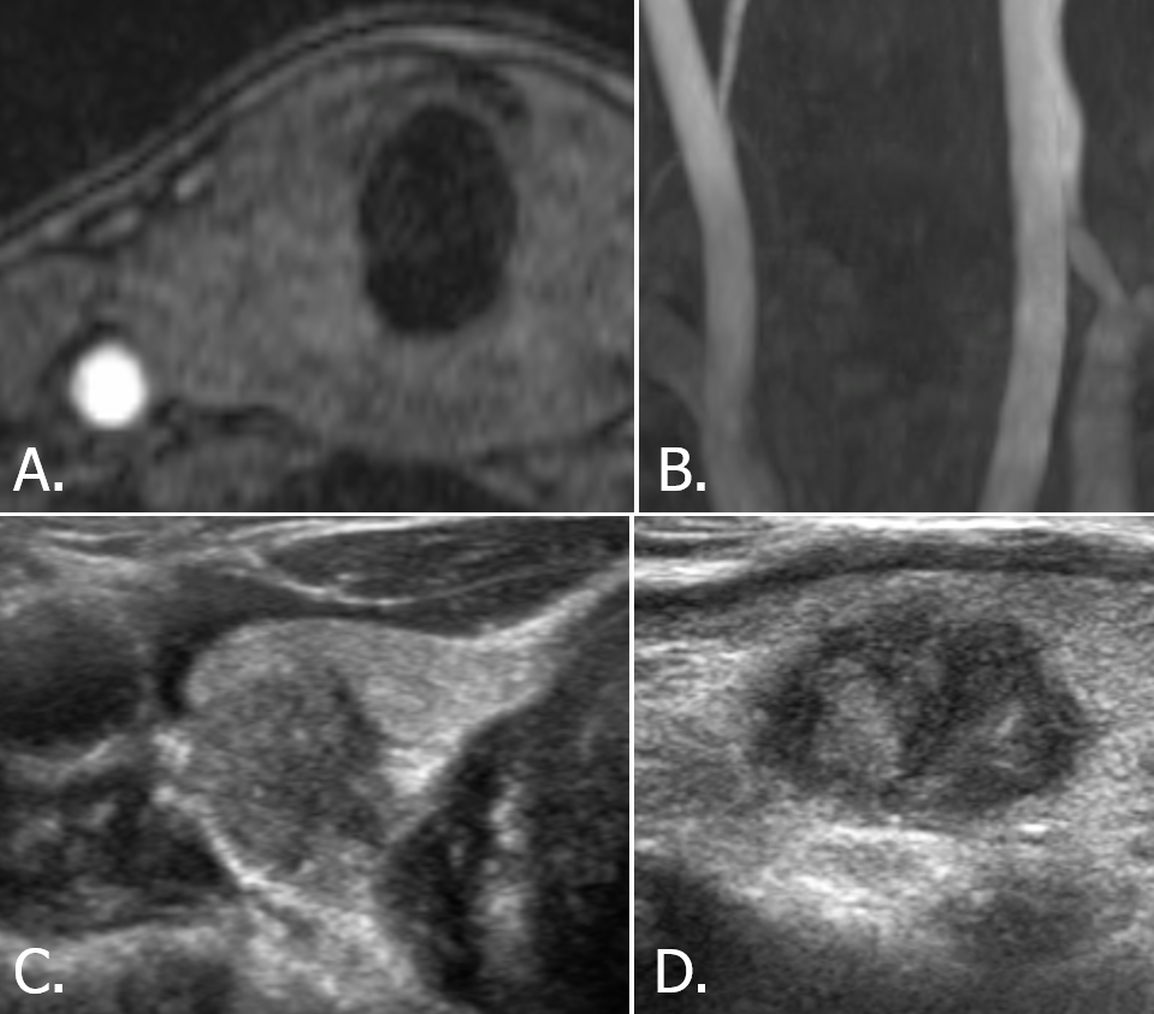

Supplement: Supplementary Figure 2 — Fifty-Two-year-old female with left papillary thyroid carcinoma. Source (A) and MIP (B) images of TOF-MR angiography show no hypersignal intense nodule in the left thyroid gland. Transverse (C) and longitudinal (D) US images of left thyroid gland demonstrates a markedly hypoechoic solid nodule with irregular/speculated margins which is classified as highly suspicious nodule (K-TIRADS, ACR-TIRADS, and EU TIRADS category 5). MIP, maximum intensity projection; TOF-MR, time-of-flight magnetic resonance; US, ultrasound; K-TIRADS, Korean Thyroid Imaging Reporting and Data System; ACR-TIRADS, ACR Thyroid Imaging Reporting and Data System; EU-TIRADS, European Thyroid Imaging Reporting and Data System; FNA, fine needle aspiration. [file Image_2.TIF]
